# Large Forest Patches Promote Breeding Success of a Terrestrial Mammal in Urban Landscapes

**DOI:** 10.1371/journal.pone.0051802

**Published:** 2013-01-02

**Authors:** Masashi Soga, Shinsuke Koike

**Affiliations:** 1 Tokyo University of Agriculture and Technology, 3-5-8 Saiwaicho, Fuchu, Tokyo 183-8509, Japan; Universität Zurich, Switzerland

## Abstract

Despite a marked increase in the focus toward biodiversity conservation in fragmented landscapes, studies that confirm species breeding success are scarce and limited. In this paper, we asked whether local (area of forest patches) and landscape (amount of suitable habitat surrounding of focal patches) factors affect the breeding success of raccoon dogs (*Nyctereutes procyonoides*) in Tokyo, Central Japan. The breeding success of raccoon dogs is easy to judge as adults travel with pups during the breeding season. We selected 21 forest patches (3.3–797.8 ha) as study sites. In each forest patch, we used infra-red-triggered cameras for a total of 60 camera days per site. We inspected each photo to determine whether it was of an adult or a pup. Although we found adult raccoon dogs in all 21 forest patches, pups were found only in 13 patches. To estimate probability of occurrence and detection for raccoon in 21 forest fragments, we used single season site occupancy models in PRESENCE program. Model selection based on AIC and model averaging showed that the occupancy probability of pups was positively affected by patch area. This result suggests that large forests improve breeding success of raccoon dogs. A major reason for the low habitat value of small, isolated patches may be the low availability of food sources and the high risk of being killed on the roads in such areas. Understanding the effects of local and landscape parameters on species breeding success may help us to devise and implement effective long-term conservation and management plans.

## Introduction

Habitat fragmentation is now a global anthropogenic cause of biodiversity decline, and it has concerned many conservation biologists for a long time [1], [2]. Previous studies [3]–[5] revealed that landscape configuration is important factors affecting species persistence in fragmented landscapes. Therefore, in fragmented landscapes, nature reserves with large, well-connected patches are preferred by many conservation biologists (e.g. [6]). Yet, current knowledge of the impact of fragmentation is largely limited to the probability of the occurrence of target species [7]–[9]. Studies that confirm factors affecting long-term species persistence (e.g. breeding success) are scarce [10], [11]. Recent empirical studies [12] have demonstrated that several species occupying fragmented landscapes eventually disappeared without further fragmentation. Therefore, it is crucial to elucidate factors affecting breeding success of the target species [13]. In this study, we asked whether both local and landscape forest cover affect the breeding success of raccoon dogs (*Nyctereutes procyonoides*) in a fragmented landscape, Tokyo, Central Japan. Raccoon dogs are an ideal model for determining breeding success because they interact with pups during the breeding season [14]. In Japan, raccoon dogs are one of the most common mammals [15], [16] and they play important roles in the forest ecosystem as seed dispersers [17] and predators [18]. Therefore, conserving raccoon dogs is crucial for sustainable management of urban ecosystems.

## Materials and Methods

### 1. Ethics Statement

In this study, we did not capture raccoon dogs. Thus, all work was legally conducted without acquiring a permit from the Tokyo Metropolitan Government or the Ministry of Environment of Japan.

### 2. Study area and sampling sites

**Figure 1:**
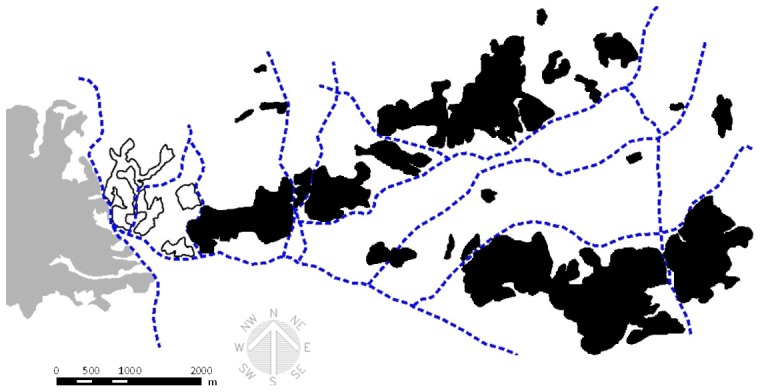
Twenty-one study forest patches (**black shaded area**)**.**

The study region was located in the southwestern Tokyo, Central Japan (Fig. 1). The western boundary of the study area was formed by a mountain range dominated by Mount Takao (599 m above sea level). The forest patches were dominated by deciduous forests comprising two oak species, *Quercus serrata* Thunb. ex Murray and *Q. acutissima* Carruthers, along with *Pinus densiflora* Sieb. et Zucc., *Abies firma* Sieb. et Zucc. and the evergreen oaks, *Q. glauca* Thunb. ex Murray and *Q. acuta* Thunb. ex Murray. The border of a forest patch was defined as any treeless belt with an open canopy of more than 300 m. In Japan, a high number of raccoon dog roadkills (about 110,000–370,000 individuals) occur every year [19]; thus, we used roadways with more than 4 lanes as borders of forest patches. A total of 21 forest patches were selected that ranged widely in size (3.3–797.8 ha) (Fig. 1).

In this study, we used forest patch area and total forest area within 600 m from a focal patch as ‘local’ and ‘landscape’ variables, respectively. Other authors [20] stressed the positive influence of %forest cover within buffers. However, in this study, such an index had no significant effects on the breeding success of raccoon dogs, so we removed it from later analyses. Both local and landscape variables were calculated from detailed aerial photographs in 2011 (Geospatial Information Authority of Japan) using ArcView geographic information system software (ver. 3.2, ESRI, Redlands, CA).

### 3. Camera trapping

**Figure 2:**
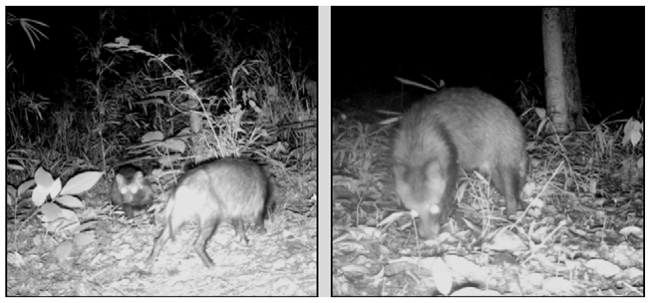
Photograph of an adult raccoon dog with pup (**left**) **and a lone adult** (**right**)**.**

The presence of raccoon dogs in 21 forest patches was assessed from June to August 2011. Raccoon dogs bear pups in May and parents and pups move together a month later. Because pups leave their parents in September, we can determine whether raccoon dogs have succeeded in breeding by taking photographs of them from June to August (Fig. 2). In each forest patch and backyard site we set infra-red-triggered cameras (National Geographic Store, 5.0 Megapixel Infrared Digital Motion-detection Camera) for a total of 60 camera days per forest patch (3 cameras × continuous 20 days); all cameras had data packs that stamped each photograph with the time and date of the event. The time delay between photographs was set to a minimum of one minute. At each site, three camera traps were widely separated and were tied to a tree 50 cm above the ground. We placed camera traps in sites where the probability of raccoon dog detection was high (e.g. animal trails). We used tainted meats as bait. We checked each camera and changed batteries once a week. We entered the photographed data into a computer recording the frame number, date, time and species for each film. The species in each photo was identified and it was determined whether only adults were present or adults with pups (Fig. 2).

### 4. Statistical analysis

To estimate probability of occurrence (*ψ*) and detection (*p*) for raccoon in 21 forest fragments, we used single season site occupancy models [21] in PRESENCE [22]. In the input spreadsheet, we inserted ‘1’ if the patch was occupied by individuals and ‘0’ if it was not occupied in a given day. In this study, we constructed nine models (Table 1) using both local and landscape variables as continuous variables. First, we held the probability of sites occupied constant, *ψ*(·), and allowed species detection to vary with each covariate, *p*(Local) and *p*(Landscape) (two models). Second, we held species detection probability constant, *p*(·), and varied*ψ*with each covariate separately, *ψ*(Local) and*ψ*(Landscape) (two models). Third, we combined *ψ*(·)*p*(Cov) model and*ψ*(Cov)*p*(·) in a*ψ*(Cov)*p*(Cov), *ψ*(Local)*p*(Local), *ψ*(Local)*p*(Landscape), *ψ*(Landscape)*p*(Local) and*ψ*(Landscape)*p*(Landscape) (four models). Also, we used a constant model, *ψ*(·)*p*(·), as a reference (one model). These nine models were ranked according to the Akaike Information Criterion (AICc) calculated by program PRESENCE. We also performed model averaging to obtain estimates and associated standard errors for each parameter of interest [23]. We repeated these procedures for both adults and pups (Table 1).

**Table 1:** AIC values and Akaike weights (w) of nine candidate models in adults and pups.

Rank	Variable (s)	AIC	ΔAIC	w *
**Adults**				
Model 1	*ψ* (.) *p*(Local)	170.51	0.00	0.288
Model 2	*ψ* (.) *p*(.)	171.46	0.95	0.179
Model 3	*ψ* (.) *p*(Landscape)	172.45	1.94	0.109
Model 4	*ψ* (Landscape) *p*(Local)	172.51	2.00	0.106
Model 5	*ψ* (Local) *p*(Local)	172.51	2.00	0.106
Model 6	*ψ* (Local) *p*(.)	173.46	2.95	0.066
Model 7	*ψ* (Landscape) *p*(.)	173.46	2.95	0.066
Model 8	*ψ* (Local) *p*(Landscape)	174.45	3.94	0.040
Model 9	*ψ* (Landscape) *p*(Landscape)	172.45	3.94	0.040
**Pups**				
Model 1	*ψ* (Local) *p*(.)	115.96	0.00	0.437
Model 2	*ψ* (Local) *p*(Landscape)	116.50	0.54	0.333
Model 3	*ψ* (Local) *p*(Local)	117.81	1.85	0.173
Model 4	*ψ* (.) *p*(Local)	121.48	5.52	0.028
Model 5	*ψ* (Landscape) *p*(Local)	123.47	7.51	0.010
Model 6	*ψ* (.) *p*(.)	124.42	8.46	0.006
Model 7	*ψ* (Landscape) *p*(.)	124.50	8.54	0.006
Model 8	*ψ* (Landscape) *p*(Landscape)	125.31	9.35	0.004
Model 9	*ψ* (.) *p*(Landscape)	126.08	10.12	0.003

* Akaike weights.

## Results and Discussion

In total, 443 photographs were taken. Adult raccoon dogs were observed in all forest patches. The best model (Model 1 in adults) suggested that detection rates of adults were 61.9 ± 5.9 (mean ± SE) %. Although the model *p*(Local) was better than the reference model (*ψ*(·)*p*(·)), their AIC differences were only 0.95 (Table 1). Also, including the landscape variable in model never improved model fit. These results suggest that occurrence probability of adults were not explained by both local and landscape variables.

Unlike adults, pups did not occur in all forest patches. We detected pups at 61.9% (13 of 21) patches. The best model (Model 1 in pups) suggested that 65.6∓12.1% of patches were occupied by pups, with a detection rate of 28.5∓5.5%. By including the local variable in our estimates of both occupancy and detection probability, *ψ*(Local) and *p*(Local), we were always able to explain the detection probability of pups more explicitly compared to the reference model (Table 1). Based on model averaging, the occurrence probability of raccoon dogs increased strongly with the local variable (i.e. forest patch area; Table 2). The 95% CI of parameter estimates of the local variable did not include zero (Table 2). On the contrary, landscape variable never improved model fit (Table 1).

**Table 2:** Model-averaged estimates for parameters of the site occupancy models of pups.

Parameters	Coefficients	SE	Lower 95% CI	Upper 95% CI
intercept	0.55	0.04	0.47	0.52
local variable	0.20	0.03	0.15	0.39
landscape variable	−0.01	0.03	−0.06	0.04

These results suggest that patch area positively affects the breeding success of raccoon dogs. In Japan, although raccoon dogs depend strongly on forests for foraging and breeding [23], they are considered to be insensitive to urbanization [24]. However, our findings indicate that the long-term survival of several populations of raccoon dogs, especially those in small and isolated patches, is not guaranteed.

There are several possible reasons why we did not observe pups in small patches. First, they need a relatively large habitat area for their daily home range [14]. Second, in small forest patches, the abundance of ground beetles, which are their main food source [25], was markedly decreased by synergistic interactions between area loss and edge effects [26]. The breeding success of vertebrates generally depends on the abundance of food resources [27]. However, in this study, the effect of landscape variable on reproductive success was not significant and weak. One possible reason of this is that the matrix environments surrounding our patches were completely urbanized. In urban areas, traffic roads are thought to have negative effects on the ability of species to migrate between patches [28]. Therefore, long-distance movement across the urban matrix is also associated with a high roadkill risk [16]. In fact, the annual raccoon dog roadkill rate in Japan is high and such accidents have a strong impact on the population dynamics and persistence of a species [17].

Determination of the effects of landscape parameters on species breeding success may help us to devise and implement effective long-term conservation and management plans. Clarifying how we should arrange many natural forest patches in fragmented landscapes before urbanization proceeds is crucial for preventing biodiversity loss worldwide and for managing forest ecosystems in urban landscapes.
